# Feasibility of open chest management with modified negative pressure wound therapy immediately after cardiac surgery

**DOI:** 10.1093/icvts/ivac041

**Published:** 2022-03-08

**Authors:** Hiroshi Kurazumi, Ryo Suzuki, Ryosuke Nawata, Toshiki Yokoyama, Sarii Tsubone, Yutaro Matsuno, Akihito Mikamo, Kimikazu Hamano

**Affiliations:** Division of Cardiac Surgery, Department of Surgery and Clinical Science, Yamaguchi University Graduate School of Medicine, Yamaguchi, Japan

**Keywords:** Delayed sternal closure, Open chest management, Negative wound pressure therapy

## Abstract

**OBJECTIVES:**

To evaluate the feasibility of open chest management with our modified negative pressure wound therapy immediately after cardiac surgery as a therapy for atypical tamponade.

**METHODS:**

Open chest with modified negative pressure wound therapy was performed immediately after cardiac surgery. The surface of the heart and the vessels were covered with non-adherent siliconized gauze. The sternal halves were stented using edge-cut disposable syringes to maintain a larger mediastinal cavity. Approximately 45 mm of distance was kept between the sternal edges. A trimmed sterile polyvinyl foam sponge was inserted into the mediastinum, the entire wound was sealed and negative pressure (−50 to −75 mmHg) was applied using a suction generator. Delayed chest closure was performed in a standard manner once the haemodynamic status was stabilizsed.

**RESULTS:**

The mortality rate was 3/15 (20%) patients. Deep sternal wound infection occurred in 1/15 (6.7%) patients. Five patients were extubated during the open chest management. Sternal closure was delayed for median of 3 days after the initial surgery. There was no incidence of bleeding complications or need for additional haemostatic procedures.

**CONCLUSIONS:**

Negative pressure wound therapy performed immediately after cardiac surgery was feasible in our small number of patients.

**Clinical registration number:**

Study ID: 2020-149.

## INTRODUCTION

Prolonged surgery, cardiopulmonary bypass or cardiac arrest can lead to myocardial, mediastinal and pulmonary oedema. In these situations, sternal closure in the immediate postoperative period can accentuate haemodynamic instability, such as hypotension, low output syndrome and intractable arrhythmias. In 1989, Kay *et al.* [1] described atypical tamponade resulting from haemodynamic impairment after open-heart surgery. Open chest management (OCM) and delayed sternal closure after cardiac surgery are therapeutic options for patients with severe cardiac impairment after surgery. Herein, we report the application of modified negative pressure wound therapy (NPWT) immediately after cardiac surgery for the management of atypical tamponade.

## PATIENTS AND METHODS

### Statement of ethics

Informed consent was obtained from the patients’ family members, such as their husband, wife, sons or daughters, who were responsible for making decisions on the patients’ behalf since they were under general anaesthesia. This retrospective study was approved by the Institutional Review Board of the Yamaguchi University Hospital (Study ID: 2020-149). The study was conducted in accordance with the Declaration of Helsinki.

### Study design and population

This was a retrospective study of cardiac and thoracic aortic surgeries performed in adults in our hospital’s cardiac division between January 2013 and June 2021. During this period of observation, 1407 eligible procedures were performed. In all cases, procedures were performed under standard general anaesthesia, cardiopulmonary bypass and cardioplegia administration. Among these cases, OCM with modified NPWT was performed immediately after surgery in 15 (1.1%) cases. Conventional OCM was not performed in any of the 1407 cases.

### Indication for open chest management

In all cases, standard chest closure was attempted. Once the sternum was fixed, haemodynamic parameters, including blood pressure, heart rate, mixed venous oxygen saturation and new onset of arrhythmia, were assessed. If the haemodynamic status after sternum fixation was considered unstable, OCM using modified NPWT was applied.

### Technique and maintenance of open chest management with modified negative pressure wound therapy

**Figure 1:**
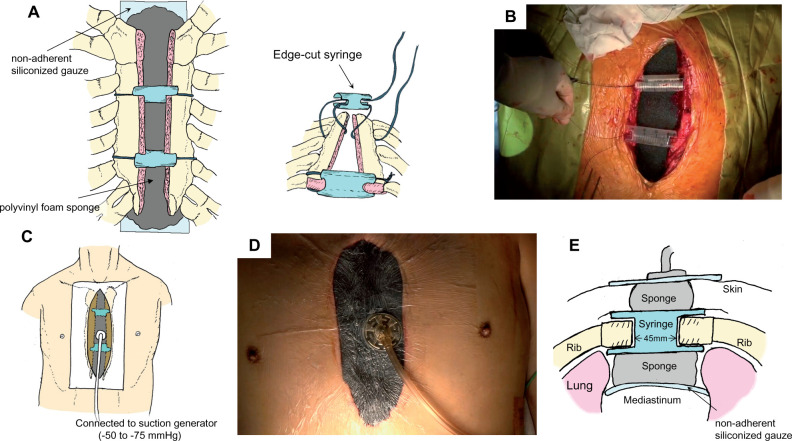
Schematic drawings and intraoperative images of the open chest management with negative pressure wound therapy applied immediately after cardiac surgery. (**A**) The sternal halves were stented using modified 20-ml syringes and a sterile polyvinyl foam sponge covered the mediastinum; the corresponding intraoperative images are shown in (**B**). (**C**) Negative pressure was applied using a suction generator; the corresponding intraoperative images are shown in (**D**). (**E**) The cross-section of this therapy.

A schematic representation of the technique, with corresponding intraoperative images, is shown in Fig. 1. We aimed for efficient intraoperative management of bleeding. The sternal halves were stented using 1 or 2, edge-cut, disposable 20-ml syringes to maintain a larger mediastinal cavity. Approximately 45 mm of distance was kept between the sternal edges using syringes. Zero monofilament sutures were used to anchor the syringes in the inter-sternal space to avoid migration. Bleeding from the sternal edges including the periosteum and bone marrow were controlled with coagulation haemostasis using electric scalpel and argon beam coagulator. Haemostatic materials (Surgical™ Nu-Knit™, Johnson and Johnson Medical, Arlington, TX, USA) were applied along the sternotomy line if bleeding from the sternal bone marrow was observed. Once bleeding was controlled, a sterile polyvinyl foam sponge was trimmed to the appropriate size to cover the wound and was inserted into the mediastinum. The surface of the heart and vessels was covered with non-adherent siliconized gauze (TREX Gauze, Fuji Systems Corporation, Tokyo, Japan) to avoid adhesion between the sponge and organs in the mediastinum. The entire wound was sealed with a transparent dressing and negative pressure was applied using a suction generator dedicated for NPWT (V.A.C.^®^ Therapy, KCI, West San Antonio, TX, USA or RENASYS™, Smith and Nephew, Watford, UK). Negative pressure was maintained between -50 and -75 mmHg during OCM. Video clip for details of our technique is available online (Video 1).

### Intensive care unit management

All patients were treated in the intensive care unit after the surgery. Once the respiratory status was stable, patients were extubated and managed under spontaneous breathing. Intravenous antibiotic therapy, initiated during the surgery (Cefazolin, 1 g every 3 h), was continued for 24 h post-surgery (Cefazolin, 1 g every 12 h). Haemodynamic status was monitored using a Swan–Ganz catheter, inserted via the right internal jugular vein.

### Timing and technique of delayed sternal closure

The negative wound dressing was removed in the operating theatre to allow haemodynamic assessment and inspection for mediastinal oedema, 2 or 3 days after the cardiac surgery. The mediastinum was irrigated using warm saline (1000 ml) and the clots were removed. Temporary re-approximation was performed to evaluate tolerance to chest closure. If the haemodynamic condition was stable, the sternum was re-fixed using 6 sternal wires, followed by closure of the major pectoris fascia and the skin, in layers. If the haemodynamic status remained unstable, OCM was continued.

### Analysis

Continuous non-parametrical data were expressed as median (IQR). Categorical variables are expressed as a count and percentage. The following clinical outcomes were described: patient characteristics, operative parameters and clinical outcomes.

## RESULTS

The preoperative patient characteristics, intraoperative parameters and clinical outcomes are shown in Table 1. The indications for surgery were as follows: 6 cases of thoracic aortic surgery for aortic dissection or aneurysm; 2 cases of infarct exclusion for ventricular septal perforation; and 2 cases of mitral valve surgery for mitral regurgitation. Bentall procedure was performed in the other 5 cases for the following reasons: infective endocarditis and aortic root abscess; left ventricular assist device implantation for fulminant myocarditis; myectomy for biventricular outflow obstruction; coronary artery bypass grafting for angina pectoris; and haemostasis for iatrogenic cardiac injury. The indications for OCM were hypotension and haemodynamic instability in 14 patients and fatal arrhythmia after chest closure in 1 patient. Haemostatic material (Surgical Nu-Knit) for bleeding from the sternal edge was used in 2 patients. The median amount of drainage to the NPWT suction device was 900 ml/day in the first 24 h and 200 ml/day in the second 24 h. There was no occurrence of cardiac or great vessel injury and no additional haemostatic procedure was required during this therapy. The in-hospital mortality rate was 20% (3/15 patients), with mortality caused by non-occlusive mesenteric ischaemia, low output syndrome and respiratory failure in 1 patient each. There was 1 case of deep sternal wound infection caused by *Serratia marcescens*. The infection developed on postoperative Day 8, 6 days after sternal closure, and was successfully treated using conventional NPWT for mediastinitis and intravenous antibiotic therapy.

**Table 1:** Patient characteristics, operative parameters and clinical outcomes

**Variables**	
**Patient factors**	
Age, years	73 (68–80)
Male, *n* (%)	9 (60)
**Operative factors**	
Emergent, *n* (%)	10 (67)
Operative time, min	590 (502–953)
CPB time, min	331 (189–458)
Cardiac arrest time, min	216 (136–335)
Intraoperative balance, ml	+8624 (+4312 to +13954)
IABP support, *n* (%)	6 (40)
ECMO support, *n* (%)	5 (33)
**Clinical outcomes**	
In-hospital death, *n* (%)	3 (20) 1 before DSC, 2 after DSC
Duration of open chest management, days	3 (3–5)
Refreshment of NPWT, times	0 (0–2)
Amount of drainage, ml/24 h (First 24 h)	900 (550–1900)
(Second 24 h)	200 (50–550)
Bleeding complication, *n* (%)	0
Time to extubation, days	5 (2–10)
Extubation during OCM, *n* (%)	5 (33)
Prolonged ventilation (>3 days), *n* (%)	9 (60)
Tracheotomy, *n* (%)	6 (40)
Superficial SSI, *n* (%)	0
DSWI, *n* (%)	1 (6.7)
Hospital stay, days	33 (17–114)
Discharged home, *n* (%)	8 (53)
Newly required ageing care home, n(%)	4 (27)

CPB: cardiopulmonary bypass; DSC: delayed sternal closure; DSWI: deep sternal wound infection; ECMO: extracorporeal membrane oxygenation; IABP: intra-aortic balloon pumping; NPWT: negative pressure wound therapy; OCM: open chest management; SSI: surgical site infection.

## DISCUSSIONS

OCM after cardiac surgery is used as a lifesaving manoeuvre for severely impaired hearts [1–3]. After sternal reopening, previous studies have used temporary chest skin closure using a sterile ePTFE membrane or pericardial sheet of xenograft, silicone sheet or latex [2, 3]. We chose to combine NPWT during OCM for patients with severe impairment in cardiac function after cardiac surgery in adults owing to the potential advantages of this novel, which include maintaining the sterile condition of the mediastinum and stabilizing the chest wall, even under OCM. NPWT was originally applied for the treatment of refractory wound infections. NPWT uses a fixed negative pressure of -100 to -125 mmHg, applied to the wound, resulting in drainage of the wound fluid and discharge. In the cardiovascular field, the efficacy of NPWT for the treatment of mediastinitis after cardiac surgery is well-established [4]. The main concern related to OCM is bacterial contamination of the open anterior mediastinum. NPWT focuses on drainage of the mediastinal space. Conventional OCM may allow bacterial contamination from the skin flora to the mediastinum cavity. NPWT during OCM may relieve cardiac compression and prevent bacterial contamination. Another potential advantage of NPWT is the decrease in chest wall fragility, stabilizing sternal mobility which can lead to lesser pain and better respiratory function. In previous studies, patients were sedated and ventilated during OCM until chest closure. However, it is well known that prolonged ventilation and sedation increase the risk for atelectasis and pneumoniae. In our study group of 15 patients, successful extubation was achieved during OCM in 5, with better respiratory function maintained in all 5 patients. In terms of complications, deep sternal wound infection developed in 1 patient who was severely deconditioned. In this case, the inflammatory mediastinitis was caused by *S. marcescens* bacteria, which was different from his skin flora (*Bacillus* spp.). Therefore, contamination from the skin flora to the mediastinum was unlikely in this case.

One potential disadvantage of NPWT after cardiac surgery is that the negative pressure may lead to exacerbation of postoperative bleeding and injury of the heart or great vessels. To minimize this risk, we aimed for efficient intraoperative management of bleeding. Bleeding from the sternal edge is a major problem during OCM as the sternal free-edge bleeds easily. We controlled bleeding from the sternal edge via coagulation haemostasis using electrocautery and argon beam coagulator or a haemostatic material (Surgical Nu-Knit), which is oxidized regenerated cellulose widely used as a topical haemostatic agent [5], attached to the sternotomy line. We used this material in 2 patients. The bleeding from the sternum or mediastinum after the surgery may lead to clogging of the NPWT sponges. However, the sponge was not clogged in our 15 patients. Injury of the innominate vein or right ventricle may also occur at the time of sponge removal because of adhesion to the sponge. Therefore, we covered the surface of the heart and vessels with non-adherent siliconized gauze to avoid adhesion between the sponge and organs in the mediastinum (Fig. 1E). Furthermore, we applied lower negative pressure (-50 to -75 mmHg) compared to standard NPWT (-100 to -125 mmHg). There was no incidence of uncontrollable bleeding or injury to the great vessels or heart injury in our study group.

Our study has 2 major limitations, which need to be acknowledged. First is the absence of a control group that underwent conventional OCM for comparison. Second, this was a single-centre study assessing a small cohort of patients.

## CONCLUSIONS

OCM with modified NPWT immediately after cardiac surgery was feasible in our small number of patients. Studies on further cumulative cases are warranted.

**Conflict of interest:** none declared.

### Data availability statement

The data underlying this article will be shared on reasonable request to the corresponding author.

### Reviewer information

Interactive CardioVascular and Thoracic Surgery thanks Ulrich Otto von Oppell, Akimasa Morisaki, Stefan Thelin and the other anonymous reviewers for their contribution to the peer review process of this article.

## ABBREVIATIONS

NPWT: Negative pressure wound therapy
OCM: Open chest management
